# Miniature Ultrasonic Spatial Localization Module in the Lightweight Interactive

**DOI:** 10.3390/mi15010071

**Published:** 2023-12-29

**Authors:** Lieguang Li, Xueying Xiu, Haochen Lyu, Haolin Yang, Ahmad Safari, Songsong Zhang

**Affiliations:** 1 School of Microelectronics, Shanghai University, Shanghai 200444, China; 21723791@shu.edu.cn (L.L.); xueying.xiu@chiponsensing.com (X.X.); yhl1765@shu.edu.cn (H.Y.); 2 Department of Materials Science and Engineering in Rutgers, The State University of New Jersey, Piscataway, NJ 08854, USA; hl827@scarletmail.rutgers.edu (H.L.); safari@soe.rutgers.edu (A.S.)

**Keywords:** blind spot free measurement, lightweight interactive, piezoelectric micromechanical ultrasonic transducers (PMUT), spatial localization

## Abstract

The advancement of spatial interaction technology has greatly enriched the domain of consumer electronics. Traditional solutions based on optical technologies suffers high power consumption and significant costs, making them less ideal in lightweight implementations. In contrast, ultrasonic solutions stand out due to their lower power consumption and cost-effectiveness, capturing widespread attention and interest. This paper addresses the challenges associated with the application of ultrasound sensors in spatial localization. Traditional ultrasound systems are hindered by blind spots, large physical dimensions, and constrained measurement ranges, limiting their practical applicability. To overcome these limitations, this paper proposes a miniature ultrasonic spatial localization module employing piezoelectric micromechanical ultrasonic transducers (PMUTs). The module is comprised of three devices each with dimension of 1.2 mm × 1.2 mm × 0.5 mm, operating at a frequency of around 180 kHz. This configuration facilitates a comprehensive distance detection range of 0–800 mm within 80° directivity, devoid of blind spot. The error rate and failure range of measurement as well as their relationship with the SNR (signal-to-noise ratio) are also thoroughly investigated. This work heralds a significant enhancement in hand spatial localization capabilities, propelling advancements in acoustic sensor applications of the meta-universe.

## 1. Introduction

Traditional virtual interaction modules predominantly utilize visual sensors to emulate the human visual system’s capacity to accurately and swiftly track a moving hand and efficiently ascertain the dynamic configuration of its underlying skeleton [1,2,3]. Visual sensor-based spatial localization approaches possess inherent advantages, such as directly deriving an object’s approximate spatial position from images and garnering motion insights through continuous image processing. However, these approaches also grapple with significant challenges. A crucial limitation is their dependency on line-of-sight observations for object localization, resulting in potential disruptions due to the temporary disappearance of some or all parts of the localized object from the scene. This susceptibility also implies a compromised ability to effectively recognize smaller localized objects within expansive captured spaces. Moreover, visual sensors exhibit reduced resilience to variations in ambient lighting, performing sub-optimally under robust sunlight conditions [4]. Additionally, acquiring depth-of-field information typically necessitates the deployment of multiple visual sensors, thereby escalating costs and energy consumption [5], while only yielding limited depth-of-field insights. In contrast, ultrasonic sensors enhance localization by processing echo signals reflected from localized objects. This approach endows ultrasonic sensors with enhanced continuity, minimizing localization interruptions as long as interacting objects stay within the sensors.

Ultrasound sensors offer significant advantages in virtual interactions due to their propagation characteristics. Their broader propagation range facilitates effective object recognition, coupled with low energy consumption and robust anti-interference capabilities [6,7]. These attributes effectively address the limitations inherent in optical solutions. However, early ultrasound sensors, primarily based on traditional piezoelectric ceramics, encounter challenges such as larger sizes, higher costs, and limited functionality, hindering their integration into existing virtual interactive devices [8,9,10].

The emergence of microelectromechanical systems (MEMS) technology signifies a revolutionary progression in this field, fostering extensive research into micromechanical ultrasonic transducers (MUTs) [11,12]. MUTs, characterized by their compact size, cost-efficiency, and low power consumption, are progressively superseding conventional piezoelectric transducers in spatial localization applications [13,14,15].

Micromechanical ultrasonic transducers (MUTs) are categorized into two types: capacitive micromachined ultrasonic transducers (CMUTs) and piezoelectric micromachined ultrasonic transducers (PMUTs). The signal transmission efficiency of CMUTs inherently relies on the inversely proportional relationship with the capacitor gap. This correlation necessitates the adoption of sub-micrometer-scale capacitor gaps in CMUTs [16], facilitating intricate fabrication processes but also limiting the linear vibration amplitudes [17]. Conversely, PMUTs operate free from the constraints of high DC voltages for effective coupling [18,19], and they do not impose geometric restrictions on mechanical displacement. PMUT, with its compact size, cost-effectiveness, low power consumption, and superior acoustic impedance matching, emerges as an exceptionally suitable choice for advancing meta-universe developments in spatial localization [20,21,22].

Over the last decade, there has been a surge in global research interest focusing on the application of ultrasound transducers for spatial localization. In 2015, Agus Naba et al. proposed an ultrasound solution based on a conventional piezoelectric bulk transducer with resonant frequency of 40 kHz, achieving high-precision measurements with 0.5 mm accuracy from distances ranging between 30 mm and 100 mm [23]. In the same year, Przybyla et al. introduced an ultrasonic module that utilized a 220 kHz resonance frequency PMUTs array, capable of measuring ranges from 45 mm to 1 m within plus and minus 45° [24]. While the measurement range of this solution was commendable, its application was hampered by a high system complexity and inability to be devoid of blind spot measurements. In 2021, Z. Shao et al. achieved a measurement distance of 1.5 m as well as a sound field angle of 90 degrees with an array of PMUTs [25], Yihsiang Chiu et al. utilized two PMUTs separately for transmitting and receiving. This system achieved 0.63 mm accuracy within a range of 100 mm to 500 mm [7]. Zhihao Tong et al. proposed a sensing skin for obstacle avoidance, employing three 3 × 3 PMUT arrays with a resonant frequency of around 115 kHz. This system, arranged linearly and spaced 23 mm apart, managed to achieve a measurement range of 125 to 300 mm within ±30°, discerning direction based on the time-of-flight (ToF) difference between the two receiving ends [26]. Despite these advancements, there exists potential for further refinement in terms of measurement distance and range. While the studies mentioned above have propelled advancements in the utilization of ultrasonic sensors for spatial positioning, they have yet to overcome key challenges. These include the use of excessively large module sizes [27,28,29] and the persistence of blind spots in measurements. Moreover, a discernible gap in the literature persists, with insufficient comprehensive research dedicated to investigating the underlying causes of failures in the measurement range and angles of ultrasound modules applied in spatial locations.

In this study, the large module sizes and the persistence of blind spots in prior ultrasonic interaction solutions were addressed, with careful calibration of the measurement distance and angle error. A compact ultrasonic module was developed, comprising three PMUT devices, each measuring 1.2 mm × 1.2 mm × 0.5 mm. This innovative module boasts capabilities such as long-distance, blind-spot-free measurements ranging from 0 to 800 mm, and a comprehensive measurement scope extending up to ±40°. A thorough analysis was conducted to meticulously examine the error rates and limitations inherent in the device’s distance and angle measurements, aiming to unveil their fundamental causes. Solutions to problems such as cumbersome sizes and prevalent blind spots in ultrasonic modules, thereby catalyzing advancements in the application of ultrasonic sensors for lightweight interaction implementations.

## 2. Structure and Characterization of PMUT

**Figure 1:**
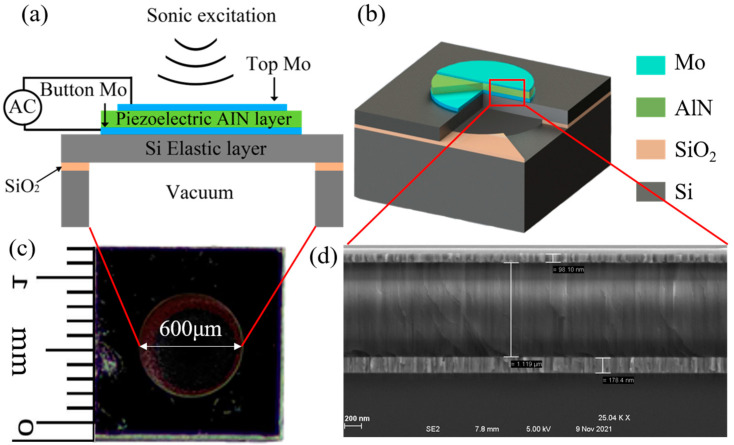
(**a**) Cross-sectional view and excitation schematic of an individual PMUT. (**b**) Lateral 3-D schematic of a PMUT in this design. (**c**) Physical image of PMUT back cavity. (**d**) Zoomed-in view HIM image of the cross-sectional structure of the released membrane.

Figure 1a,b depict schematic and three-dimensional structures of the scandium aluminum nitride (Sc_0.2_Al_0.8_N)-based piezoelectric micromachined ultrasonic transducer (PMUT). This meticulously designed transducer comprises sequentially layered components: a top electrode made of molybdenum, an ScAlN piezoelectric layer, a bottom molybdenum electrode, a silicon support layer, and a silicon substrate that encompasses a back-etched cavity. In this innovative assembly, a vibratory membrane is released through the cohesive stacking of the electrode and piezoelectric layers along with the support layer. This membrane is subtly liberated by back etching from the substrate, facilitating precise vibrational functionalities. When subjected to alternating current (AC) voltage excitations across the upper and lower electrodes, the piezoelectric layer undergoes deformation. This action, attributed to the piezoelectric effect, propels the membrane into a state of vibration, enabling it to emit acoustic waves with refined efficacy. Figure 1c reveals a microscopic inspection of the device’s back cavity, showcasing its meticulously crafted square shape, each side measuring 1.2 mm, complemented by a back cavity diameter of 600 μm. In Figure 1d, an augmented cross-sectional view of the dissected PMUT membrane is presented, emphasizing the meticulously measured thickness of each constituent layer. The measurements reveal a top electrode thickness of 98.1 nm, a substantial piezoelectric AlN layer measuring 1.119 μm, and a bottom electrode with a thickness of 178.4 nm.

**Figure 2:**
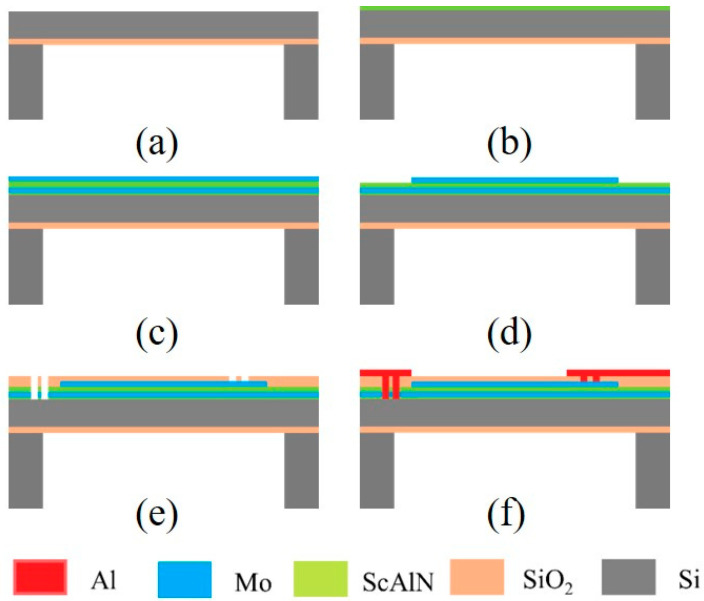
Fabrication process flow of the ScAlN-based PMUT. (**a**) customizing a cavity-SOI substrate, (**b**) depositing AlN seed layer, (**c**) multiple layers sputtering, (**d**) etching top electrodes, (**e**) depositing and etching a SiO_2_ insulation layer via holes, and (**f**) deposition and patterning of Al metal layer for electrical connections and to form bonding pads.

The fabrication process of the PMUT starts from a silicon-on-insulator (SOI) wafer, silicon support layer thickness of 4 μm (Figure 2a). Prior to the deposition of Mo/ScAlN/Mo stack, an AlN seeding layer is deposited (Figure 2b). Next, 0.1 μm Mo/1 μm ScAlN/0.2 μm Mo stack on the ScAlN seeding layer by using magnetron sputtering deposition (Figure 2c). The top Mo layer is formed by plasma etching (Figure 2d). An oxide layer is deposited to form the isolation layer, followed by the etching of SiO_2_ and ScAlN to pattern the via opening for the top and bottom electrodes (Figure 2e). Deposition and patterning of aluminum (Al) leads and bonding pads (Figure 2f).

**Figure 3:**
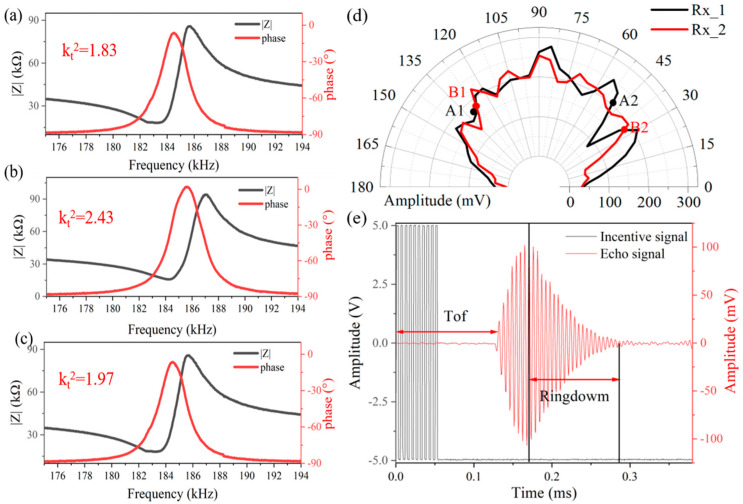
(**a**,**c**) The impedance-frequency spectrum of the PMUT device at the Rx in air. (**b**) The impedance-frequency spectrum of the PMUT device at Tx in air. (**d**) Analytical and experimental results of the directivity of Tx1 (black) and Tx2 (red). (**e**) Acoustic time-domain characterization: acoustic measurements use a designed PMUT and a calibrated reference microphone. The PMUT is excited by 10-cycle square continue wave with driving voltage of 10 Vpp.

Figure 3a–c shows the electric properties of the fabricated PMUT. The impedance and phase were measured by an impedance analyzer (Keysight E4990A). The results show that the resonant frequencies of the PMUT in air for the three devices measured by the impedance analyzer are 183.025 kHz, 184.281 kHz, and 183.093 kHz, respectively. The electromechanical coupling factor ($k_{t}^{2}$) can be estimated by approximating the following equation:

(1) $$k_{t}^{2}=\frac{C_{m}}{C_{m}+C_{o}}$$

where $C_{m}$ is the equivalent capacitance of the PMUT, and $C_{o}$ is the parasitic capacitance of the PMUT. The calculated $k_{t}^{2}$ is 1.83%, 2.43%, and 1.97%, respectively.

To characterize the acoustic directivity at the receiving end, the PMUT was centrally affixed to a custom rotary displacement stage. Positioned 150 mm away from the device, the baffle was methodically moved in 5° increments. Figure 3d presents both the analytical and experimental results of the PMUT devices’ directivity, operating under a 5-volt condition.

It can be seen that the acoustic field angles of the two devices are very close to each other. The results indicate that the main-lobe half-power beamwidth (HPBW), denoted as θ_−3_ dB, of Tx1 is 83°, while for Tx2 it is 94°. By considering the intersection of the half-power beamwidths of the two receivers, the half-power acoustic field angle of this ultrasound module is determined to range between 48° and 127°

Figure 3e shows the echo signals captured by the microphone, positioned 20 mm away from the device, when a 10 Vpp, 10-burst square wave excitation signal is administered through the signal generator. The time of flight (ToF), which is also the interval between the excitation and the first echo, can be utilized to compute the distance between the device and the object.

The output pulse is shaped by the dynamics of the transducer, and the mechanical energy stored in the transducer dissipates as the transducer rings at the resonant frequency after the firing cycle. Assuming that the transducer is excited to full amplitude at f0, the ringing current $i_{ring}\left(t\right)$ [21]

(2) $$i_{ring}\left(t\right)=\frac{V_{TX}}{R_{M}}u\left(t-T_{TX}\right)e^{-\omega_{B}\left(t-T_{TX}\right)}\cos\left(\omega_{0}t\right)$$

where $T_{TX}$ is the transmit time and $R_{M}$ is the motional resistance. $\omega_{B}$ is the bandwidth of the device. The Equation (2) shows that the ringing current $i_{ring}$ is inversely proportional to the bandwidth, when the ringing current is too long, the bandwidth is small, it will make the primary echo and the secondary echo in the proximity of the distance in the nearer distance overlap, expanding the measurement distance of the blind spot. The device used in this article has a −3 dB bandwidth of 9.7 kHz, and in the constructed spatial localization system, without using additional algorithms, a signal of 20 mm can be measured at the closest distance.

## 3. System Principle

Ultrasonic ranging using the ToF principle is based on the measurement of the time that the acoustic echo travels from the transmitter to the object and returns to the receiver. The measured ToF *t* is related to the flight distance *L*, ultrasonic velocity *c*, distance *S* between the transmitter and receiver, and the reflection angle θ between the object and the source plane. Therefore, as long as ToF is accurately measured, ultrasonic sensors can measure the distance to the object.

**Figure 4:**
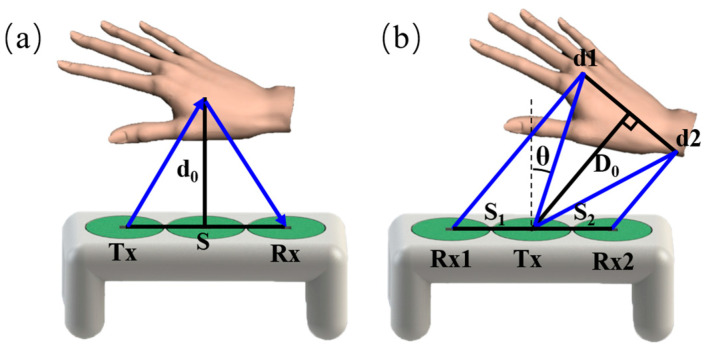
ToF principle of ultrasonic ranging as the target object is in front of the ultrasonic source with an angle of zero (**a**) and *θ* (**b**).

As shown in Figure 4a, when the object is directly in front of the ultrasonic wave source and the angle is zero, the distance *d*_0_ between the ultrasonic sensor and the object can be expressed as

(3) $$d_{0}=\frac{\sqrt{c^{2}t^{2}-s^{2}}}{2}$$

where c is the speed of sound, t is the propagation time of the ultrasonic wave, and s is the distance between the two devices.

When the angle between the object and the device is θ, distance measurement can be achieved by using one transmitter Tx and two receivers (Rx1 and Rx2). As Figure 4b, $t_{1}$ and $t_{2}$ received by Rx1 and Rx2 it can be expressed as

(4) $$t_{1}=\frac{d_{1}}{c}=\frac{\sqrt{{\left({2D}_{0}-S_{1}sin\theta \right)}^{2}+{\left(S_{1}cos\theta \right)}^{2}}}{c}$$

(5) $$t_{2}=\frac{d_{2}}{c}=\frac{\sqrt{{\left({2D}_{0}+S_{2}sin\theta \right)}^{2}+{\left(S_{2}cos\theta \right)}^{2}}}{c}$$

where $d_{1}$ are the flight distances from Tx to the object and from the object to Rx1, $d_{2}$ are the flight distances from Tx to the object and from the object to Rx2, $D_{0}$ is the measured distance between the object and Tx, $S_{1}$, and $S_{2}$ are the distances from Tx to the two receivers Rx1 and Rx2, respectively.

In Figure 4b, the flight distance $d_{1}$ of ultrasound through Tx object Rx1 is smaller than $d_{2}$ of ultrasound through Tx object Rx2. Therefore, the echoes received from Rx1 and Rx2 generate ToF differences. When the distance between Tx and Rx1 and Rx2 is the same as S, the distance $D_{0}$ and angle between the object and Tx are the same θ can be obtained from (6) and (7), such as

(6) $$D_{0}=\sqrt{\frac{c^{2}\left(t_{1}^{2}+t_{2}^{2}\right)-2s^{2}}{8}}$$

(7) $$\theta ={sin}^{-1}\left[\frac{c^{2}\left(t_{1}^{2}-t_{2}^{2}\right)}{8D_{0}S}\right]$$

## 4. Experiments

### 4.1. Space Positioning System

**Figure 5:**
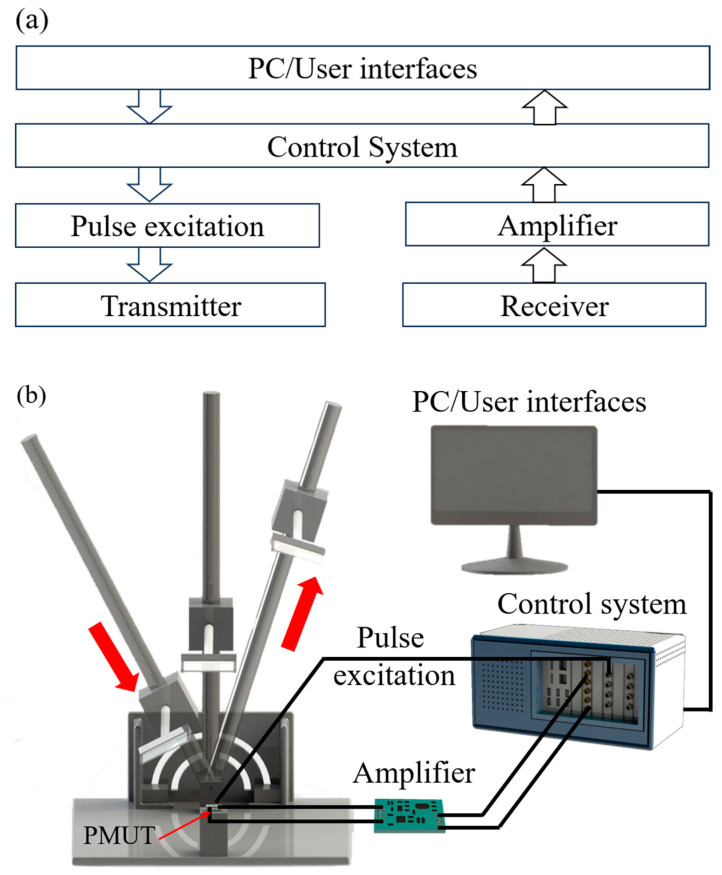
(**a**) Block diagram and (**b**) experimental setup of the ultrasonic measurement system.

The ultrasonic-based virtual interaction module discerns the position of an object by analyzing the detected echoes. It assesses the height, orientation, and angular information of the object, correlating these with the respective echo responses, utilizing a measurement system illustrated in Figure 5. The ultrasonic module is positioned at the center of a rotary displacement stage. This stage is calibrated, allowing for displacements ranging from 0 to 1000 mm in precise 1 mm increments and rotational adjustments spanning 180°, facilitated in exact 1° steps. Throughout the measurement phase, a computer-controlled waveform generator consistently produces a burst signal (183 kHz, 10 Vpp, 10 cycle), directing it towards the transmitter (Tx), while dispatching a synchronization signal to the data acquisition card. The received signals at Rx1 and Rx2 are proficiently amplified by a dedicated amplifier board and then captured by the data acquisition card. Comprehensive information pertaining to the object is extracted by processing these acquired echo signals. This setup facilitates a dynamic modification of both distance and angle between the reflective plate and the ultrasound module.

### 4.2. Distance Information

The problem derived from blind spot is solved by applying fractional Fourier transform (FrFT). FrFT is used to extract a primary echo efficiently when multiple echoes are overlapping.

In 1980, Namias described an incomplete form of the fractional Fourier transform [30], which is a generalization of the Fourier transform (FT). In 1987, McBride and Kerr published an extended analysis of the FrFT [31], on which recent work is based. The conventional Fourier transform allows signals in the time domain to be transformed into the frequency domain and vice versa, the fractional Fourier transform allows signals to be transformed into the fractional domain. The fractional domain is neither time nor frequency, but is an intermediate domain between the time and frequency domains, called the fractional domain. The difficulty in achieving blind spot free measurements lies in extracting the primary echo from a signal with multiple echoes mixed together, which can be better achieved in the fractional domain than in signal processing in the time and frequency domains.

The Fourier transform can be expressed as

(8) $$X\left(f\right)=\int_{-\infty }^{\infty }B\left(f,t\right)x\left(t\right)dt$$

where $B\left(f,t\right)$ is the transformation kernel.

(9) $$B\left(f,t\right)=exp(-j2\pi ft)$$

where t is the time and *f* is the frequency. FrFT is defined by modifying the Fourier transform kernel to the form [31,32].

(10) $$B_{\varphi \left(x,y\right)}=A_{\varphi }exp\left[j\pi \left(x^{2}\cot\left(\varphi \right)-2xy\csc\left(\varphi \right)+y^{2}\cot\left(\varphi \right)\right)\right]$$

where $A_{\varphi }$ can expressed as

(11) $$A_{\varphi }={|\sin\left(\varphi \right)|}^{\frac{1}{2}}exp\left[\frac{-j\pi \mathrm{sgn}\left(\sin\left(\varphi \right)\right)}{4}+j\frac{\varphi }{2}\right]$$

where x and y define the axes of the fraction domain. Instead of defining the fraction of the transformation as the angle φ in the interval [–*π*, *π*] radians, the new variable α is defined as the order of the transformation; α is valid in the interval [–2, 2] and is defined as

(12) $$\alpha =\frac{2\varphi }{\pi }$$

**Figure 6:**
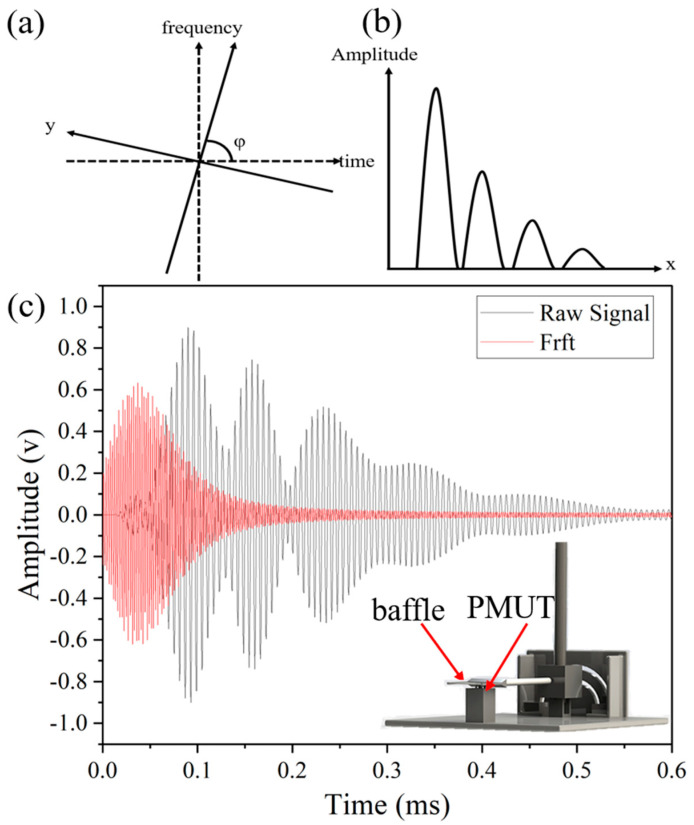
(**a**) Clockwise rotation of time-frequency plane (*t*–*f*) by angle, *φ*, through the use of the FrFT, forming a new reference plane, (*x*–*y*). (**b**) After the appropriate angle *φ* is selected, it can be seen in the fractional domain of that after the FrFT, the multiple echoes are not mixed together. (**c**) Echo signal when the baffle is right next to the device (black) and the primary echo extracted by FrFT (red).

The most relevant interpretation of the FrFT with respect to ultrasound is that the Wigner–Ville distribution of the signal is rotated clockwise in the time-frequency plane by an angle φ [32,33,34]. In Figure 6a, the time-frequency plane (t–f) is rotated clockwise by an angle φ to form a new reference plane (x–y) [31,33,35].

The process of extracting the primary echo is to first perform FrFT transformation on the signal that are aliased in the time domain, add windows to the signals in the fractional domain, extract the primary echo in the fractional domain separately, and finally carry out the inverse FrFT to transform the primary echo in the fractional domain back to the time domain. When different angles are selected, the corresponding signals in the fractional domain are also very different, and when the selected rotation angle φ is not appropriate, the signals in the fractional domain will still show a mixed state, and some of them will even be distorted. After the appropriate angle φ is selected, it can be seen in the fractional domain of Figure 6b that the multiple echoes are not aliased after the FrFT, and then the inverse FrFT transform is performed, and the primary echoes can be accurately extracted.

In order to achieve blind-free measurements, after repeated experiments, it was found that a single echo was best separated from the aliased signal in the fractional domain when the transform order *α* was 0.98. Multiple echoes are separated in the fractional domain. At this time, a window is added to the primary echo in the fractional domain, and then the inverse fractional Fourier transform is applied to the primary echo after the window is added, then the primary echo can be effectively extracted from the overlapping echo signal. Figure 6c presents a comparison between the original overlapped echo signals and the primary echo extracted by FrFT. It conspicuously reveals that the issue of phase shift, attributed to the echo overlap, has been resolved, yielding a highly pure primary echo signal. Figure 6c illustrates the experimental schematic employed in the measurement of the blind spot. In this setup, the reflection plate is positioned exceedingly close to the PMUT device, almost in contact. In such a case, the ToF of the primary echo obtained by calculating the fractional Fourier transform yields a distance of 3 mm, and therefore an error of 3 mm at distance 0 mm.

**Figure 7:**
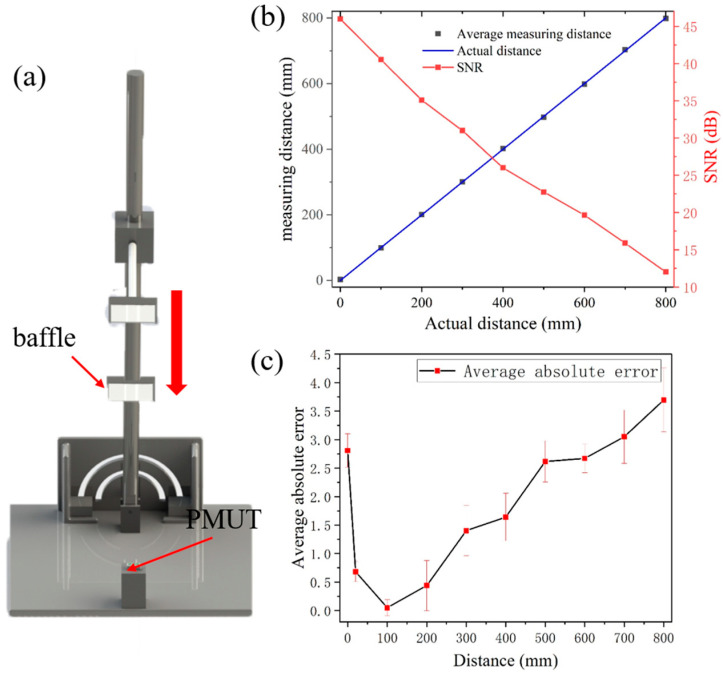
(**a**) Experimental environment for distance measurement. (**b**) The measured results of distance and SNR. (**c**) The average absolute error of measured results and standard deviation.

Figure 7a illustrates the experimental setup where the ultrasound module is centrally positioned on the rotary displacement platform, and the reflective plate is lowered from an elevated position downwards. To characterize the module’s capacity for positioning over various distances, the rotary displacement platform is gradually lowered in increments of 100 mm. The distance information is recorded and the signal-to-noise ratio (SNR) is calculated by [24]

(13) $$\mathrm{SNR}=\frac{V_{TH}^{2}}{\sigma^{2}}$$

where $V_{TH}$ is the system’s threshold and $\sigma^{2}$ is the noise variance of the system.

Figure 7b shows the comparison between the distance information collected during the descent and the actual distance. Figure 7c depicts the relative error observed from distances ranging between 0 mm and 800 mm. For distances of 0, 10, and 20 mm, the respective average relative errors recorded are 2.81 mm, 1.67 mm, and 0.68 mm. The standard deviation is from 0.17 mm to 0.56 mm. After that, there is a slight increase in error as the SNR decreases. It is evident from the observations that the absolute error maintains a consistency around 1 mm within the 200 to 400 mm range, and varies between 2 and 3 mm for distances extending from 500 to 800 mm.

### 4.3. Direction Information

**Figure 8:**
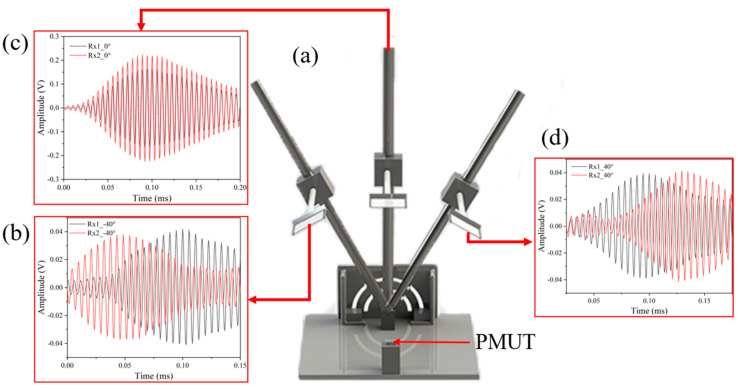
(**a**) The experimental environment of variable azimuth angles used in the experiment. (**b**) The signal waveform received by the Rx1 and Rx2 when baffle is 200 mm above Tx and −40° near the Rx1, (**c**) 0° directly above the Tx, (**d**) 40° near the Rx2.

In order to capture the orientation information of the object, data acquisition is performed for signals in different angles Figure 8a. The ultrasonic module is placed in the center of the rotary displacement platform, and the reflective plate is placed at the angles of 40°, 0°, and minus 40° between the devices, respectively. When the reflecting plate is at different angles, the ultrasonic waves will propagate to Rx1 and Rx2 with different paths, and the propagation times of the two paths are different according to Equations (4) and (5). When the distance of the ultrasonic module from the baffle is 200 mm, the baffle and the ultrasonic module are measured at three different angles. Figure 8b shows when the reflector plate is parallel to the ultrasound module, and it can be seen that there is almost no phase shift between the two receiving ends. While Figure 8c,d show the ultrasonic module and the reflector plate at angles of 40° and minus 40°, respectively, it can be seen that at 40° Tx1 is clearly ahead of Tx2, and at minus 40° Tx1 is clearly behind Tx2. From this, we can judge the left and right based on the positive and negative of the Dtof of the two receiving ends.

### 4.4. Angle Information

**Figure 9:**
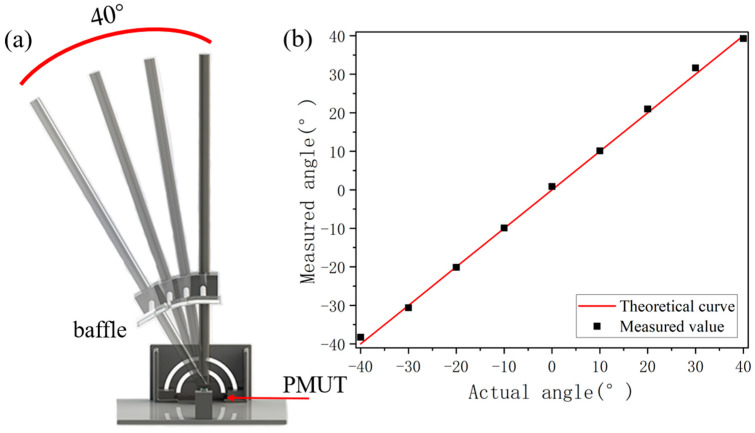
(**a**) Experimental environment for angle measurement. (**b**) The measured results of angle.

To capture the angular information of an object, data were collected at various angles at a consistent distance, and the performance was analyzed. Figure 9a illustrates the experiment setup. The ultrasound module is centrally placed on a rotating displacement platform, and the reflector plate is incrementally moved from left to right. The difference in time of flight (Dtof) is determined by calculating the point disparities in time between the two receiving ends, subsequently allowing for angle calculation via Equation (7). To characterize the range of angles at which the ultrasound module can operate, the rotating platform was gently shifted in increments of 10°. Figure 9b presents a comparison between the acquired angle information during lateral movement and the actual angles. The findings reveal that the ultrasound module described in this study can effectively localize angles within a range of plus and minus 40°.

## 5. Error Analysis

**Figure 10:**
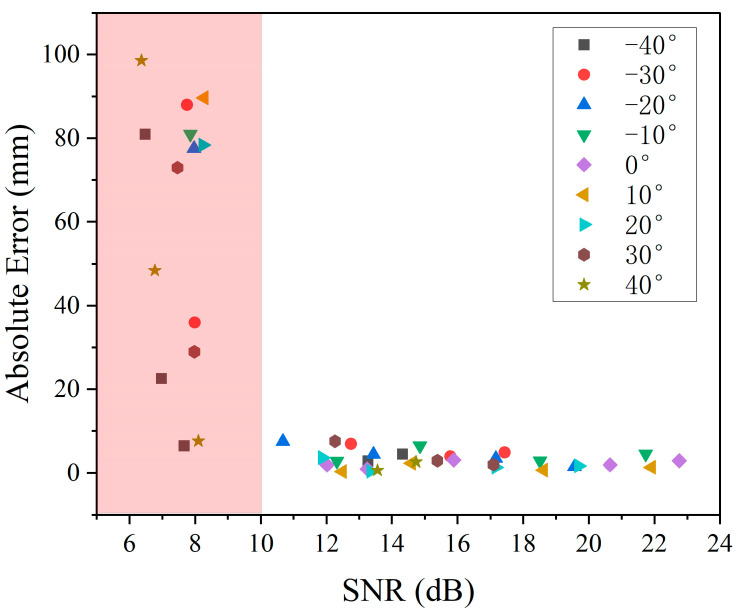
Corresponding system SNR for different distances at different angles.

In order to more precisely characterize the interactable range and accuracy during virtual interaction, the ultrasound module was fixed in the center of the rotational displacement platform. The reflector plate was moved from −40° to 40° in 10° steps. At each angle, the height of the reflective plate is varied from 100 mm to 800 mm, and Figure 10 illustrates the relationship between the signal-to-noise ratio (SNR) and the measurement error of the system. A SNR less than 10 dB results in a distance measurement error greater than 10 mm, rendering such distances effectively unmeasurable. Table 1 delineates the absolute errors corresponding to various angles and distances. Entries in the table with significant deviations from the actual values are highlighted in orange. From the data, it is discernible that the system’s maximum measurable straight-line distance is 800 mm. For distances ranging between 100 mm and 600 mm, the system can interact within an angular range of minus 40° to 40°. The maximum absolute error within this range is 5.644 mm, observed at a distance of 400 mm and an angle of 40°. As the distance extends to 700 mm, the feasible interaction angle narrows to between −30° and 30°. Within this spectrum, the most considerable absolute error recorded is 7.533 mm, occurring at a distance of 700 mm and an angle of 30°. For distances beyond 750 mm up to 800 mm, the operational angular range diminishes further, allowing interaction at angles between −20° and 20° only. At the extreme distance of 800 mm, measurable interactions are limited to instances where the system is directly facing the reflector plate, indicating the constraints of the system’s operational capacity at extended distances.

**Figure 11:**
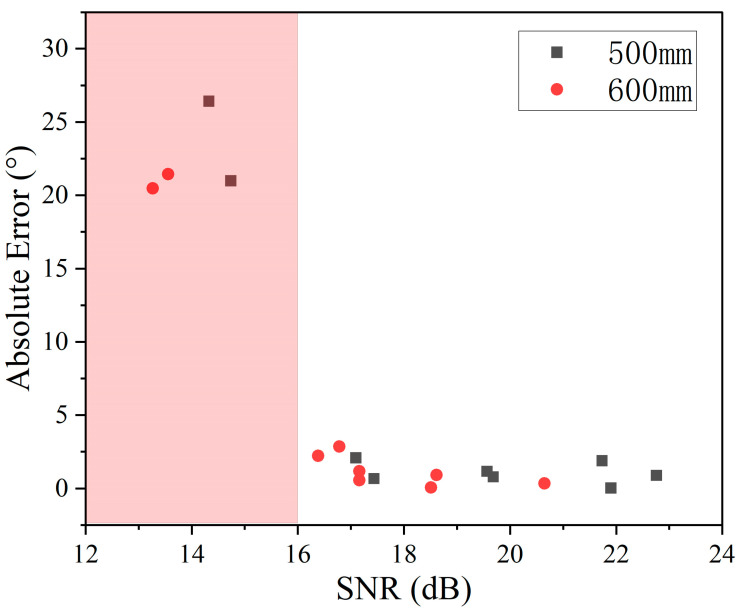
Corresponding system SNR for different angles at different distances.

From Figure 11, it can be concluded that due to the angle measurement for the sound intensity requirements are higher. A SNR less than 16 dB results in an angle measurement error greater than 3°, rendering such an angle effectively unmeasurable. As a result, the absolute error of the angle at different distances is obtained as shown in Table 2, when the straight line distance is 100–400 mm, the maximum error of the measurable angle is 2.871° at (400 mm, −30°), when the straight line distance is 500–600 mm, the maximum error of the measurable angle is 2.847° at (600 mm, −30°).

In this work, PMUT is used for spatial localization, which can achieve a blind-free measurement of 0–800 mm as well as angle measurement of ±40° and identify the direction by the positive and negative of Dtof. Table 3 lists the articles that use PMUT to do relevant measurements in the past three years, and compares them in terms of measurement distance and measurement angle. From the range of measurement distance, it can be seen that this article realizes blind-spot-free measurements in close distance, and the maximum measurement distance reaches 800 mm, and from the perspective of measurement angle, there is a progress of ±10° compared with the previous work.

## 6. Conclusions

In this work, a lightweight interactive ultrasonic module based on AlSc0.2N PMUT is proposed. This innovative module is proficient in determining the distance, direction, and angle of objects. Using a combination of TOF and fractional Fourier transform, a distance measurement of 0–800 mm was realized, solving the blind spot problem inherent in distance measurement. Directionality is ascertained by evaluating the difference in the ultrasonic wave travel distances (Dtof), allowing for precise angle determinations between the ultrasonic module and the object, thus granting the module an angular measurement range of 80°. Through an analysis linking distance and angle errors with the system’s signal-to-noise ratio (SNR), it is inferred that the system requires a minimum SNR of 10 dB for distance identification and 16 dB for angle discernment. Empirical evidence demonstrates that the proposed AlScN-based PMUT ultrasonic module, devoid of intricate system complexity, is adept at executing non-blind-zone measurements up to 800 mm, recognizing directional nuances, and accurately measuring angles up to 80°. This enhances its spatial positioning ability, optimizing the PMUT ultrasonic module for seamless integration into lightweight interactive environments.

## Figures and Tables

**Table 1:** Absolute error of distance detection at various angles.

Actual (mm)	Absolute Error of Measured Distance (mm)								
	−40°	−30°	−20°	−10°	0°	10°	20°	30°	40°
100	−1.673	−2.116	−2.426	−1.868	−0.496	−2.239	−0.276	0.374	−0.134
200	−0.425	−1.811	−2.256	−2.018	1.049	−1.392	−0.199	1.186	2.717
300	0.526	−1.374	−0.632	−0.107	0.566	−0.774	−0.272	1.124	2.669
400	0.498	0.093	0.530	0.857	2.123	1.384	1.689	4.092	5.644
500	−4.52	−4.924	−1.491	−1.166	−2.899	1.360	1.668	1.975	2.63
600	2.87	−3.937	−3.505	−3.182	−1.916	−0.656	−1.346	−2.937	0.62
700	16.46	6.953	4.482	5.805	3.071	2.332	−0.351	7.553	17.61
750	−22.53	−35.94	7.478	2.801	−0.933	0.328	3.638	−28.94	−48.38
800	−80.93	−87.94	−77.52	−90.19	−1.937	−89.67	−78.36	−72.95	−98.59

Measurable distance (green), non-measurable distance (orange).

**Table 2:** Absolute error of angle detection at various distance.

Actual (mm)	Absolute Error of Measured Angle (°)								
	−40°	−30°	−20°	−10°	0°	10°	20°	30°	40°
100	−1.273	0.018	−1.185	−0.361	−0.060	0.782	0.223	0.984	−0.424
200	1.734	−0.613	−0.127	0.093	0.871	0.107	0.969	1.649	−0.722
300	−0.186	−0.992	0.411	0.070	−0.440	0.779	0.754	1.147	0.645
400	−0.064	−2.871	−0.668	0.039	−0.904	0.434	0.677	1.545	1.372
500	26.420	−0.668	−1.165	−1.891	−0.885	−0.025	0.785	2.086	−20.990
600	20.477	−2.847	−0.566	0.052	−0.339	0.906	1.175	2.223	−21.445

Measurable angle (green), non-measurable angle (orange).

**Table 3:** Performance comparison of PMUT-based distance and angle measurements in the last three years.

Reference	Transducers	Freq. [kHz]	Range. [mm]	Angle. [°]
[7]	PMUT	96	100–500	-
[26]	PMUTs array	115	125–300	±30
[36]	PMUTs array	80	200–600	-
This work	PMUT	183	0–800	±40

## Data Availability

Data are available from the authors on request.
